# Design and Implementation of a Blue-Light-Controlled Gene-Switch System

**DOI:** 10.3390/molecules31122032

**Published:** 2026-06-10

**Authors:** Chen Li, Yuan Shi, Xinyan Jiang, Bobo Zhao, Chen Zheng, Aowei Yang, Yao Wang, Junfeng Pan, Xihui Shen

**Affiliations:** Shaanxi Key Laboratory of Agricultural and Environmental Microbiology, College of Life Sciences, Northwest A&F University, Yangling 712100, China

**Keywords:** synthetic biology, optogenetics, T7 RNA polymerase, benzoic acid

## Abstract

Synthetic biology seeks to build predictable, programmable biological systems. We developed a blue-light-inducible T7RNAP system with dual-input regulation to enable precise spatiotemporal gene control, which is vital for biomanufacturing, therapy, and microbial engineering. We optimized it by replacing RBS sequences, testing tandem T7 promoters, and evaluating split-T7RNAP variants. Expression and bactericidal efficacy were assessed via fluorescent output and real-time growth curves under blue light. RBS variants caused up to 50-fold differences in expression. Three tandem T7 promoters provided the best balance between yield and fidelity. Integration of a benzoate-responsive module enabled 4.5-fold repression at 3 mM benzoate, demonstrating effective chemical off-switching without compromising light induction. This system combines blue light precision with environmental responsiveness, offering non-invasive, on-demand activation for antimicrobial therapy or spatial bioproduction. The benzoate-triggered off-switch is especially valuable for ecological applications such as biocontainment or bioremediation, where gene expression must shut down upon detection of pollutants, for example, aromatic hydrocarbons. Its orthogonal, modular design supports context-dependent control, making it ideal for environmental biosensors, programmable probiotics, and smart antimicrobial delivery in complex ecosystems.

## 1. Introduction

Synthetic biology has emerged as one of the most transformative disciplines of the 21st century, applying engineering principles such as standardization, modularity, and programmability to biological systems. This enables the design of novel genetic circuits that perform functions not found in nature, shifting the field from passive observation to active biological engineering [1]. Its impact is already evident across diverse domains: engineered *Escherichia coli* now produce dencichine for hemostasis [2], while *Saccharomyces cerevisiae* has been optimized to synthesize ginsenoside compound K at a titer of 5.0 g/L [3,4,5] and catharanthin, a precursor to the anticancer drug vinblastin [6]. In metabolic engineering, cyanobacteria have been redesigned for enhanced carbon fixation and biofuel production [7]; in food science, synthetic biology enables the efficient biosynthesis of mogrosides, a natural and health-promoting sweetener, from *Siraitia grosvenorii* [8]. Despite these remarkable achievements, the precise spatiotemporal control of gene expression in complex metabolic networks remains a persistent challenge. Traditional regulatory tools often suffer from context-dependent noise, limited dynamic range, and promoter cross-talk, which can bottleneck metabolic flux optimization. To address these limitations, synthetic biology introduces modular, tunable, and orthogonal genetic circuits that enable precise, multi-parameter regulation of host metabolism. It is within this framework that developing robust, multi-inducer systems becomes critical for next-generation bioproduction, directly motivating the design of the triple-regulated circuit presented in this study. As the field matures, its focus is increasingly shifting from lab-scale proof-of-concept to real-world deployment, particularly in environmental monitoring, bioremediation, and point-of-care diagnostics, where gene circuits must operate autonomously, robustly, and precisely under non-laboratory conditions.

Among the most powerful tools for achieving precise spatiotemporal control in synthetic biology is optogenetics, defined as the use of light to regulate cellular processes with high specificity and minimal invasiveness. Unlike traditional chemical inducers such as IPTG or arabinose, which diffuse broadly and require hours to equilibrate, light offers micrometer spatial resolution and millisecond temporal precision, enabling fine control over gene expression, protein localization, and metabolic flux [9,10]. Optogenetic systems typically rely on light-sensitive domains from plants, fungi, or bacteria that undergo conformational changes upon illumination, thereby modulating downstream genetic circuits. Well-characterized modules include the CRY2/CIB1 system from *Arabidopsis thaliana* [11] and the VVD and Magnets systems from *Neurospora crassa* [12,13]. The pDusk/pDawn system, developed by Möglich and colleagues, uses a two-component histidine kinase-response regulator pair (YF1/FixJ) to enable red- and far-red-light-responsive gene expression [14]. In one notable example, researchers replaced the arabinose-sensing domain of the transcriptional activator AraC with the blue-light–sensitive VVD domain from *Neurospora crassa*, thereby reprogramming AraC to respond to light instead of sugar [15]. The key advantages of optogenetics include non-invasiveness, reversibility, tunability, and scalability, which make it particularly suitable for applications requiring dynamic, programmable, and environmentally compatible control.

While optogenetics provides exquisite spatial and temporal control, the transcriptional output of any gene circuit ultimately depends on the efficiency and specificity of the RNA polymerase. In synthetic biology, T7 RNA polymerase (T7RNAP) has become the gold standard for high-level, orthogonal gene expression [16]. Derived from bacteriophage T7, T7RNAP is a single-subunit enzyme that recognizes a highly specific 17 bp promoter sequence, which is rarely found in bacterial or eukaryotic genomes. Prior studies have successfully engineered blue-light-inducible split T7 RNAP systems by fusing T7 RNAP fragments to photosensitive domains such as the nMag/pMag [13] and the VVD photosensor [17]. These systems enable precise transcriptional control in response to blue light and serve as the conceptual foundation for our dual input design. This orthogonality allows T7RNAP to drive transcription of target genes without interfering with endogenous cellular processes. Moreover, T7RNAP is known for its exceptionally high transcriptional activity. Recent advances have further enhanced its utility by integrating it with CRISPR interference (CRISPRi) systems [18]. Researchers have designed single-guide RNA (sgRNA) targeting the PT7/LacO1 promoter, enabling precise repression of leaky expression through dCas9-mediated steric hindrance [19]. This combination provides a powerful strategy for achieving both high expression levels and tight regulatory control, which is a key requirement for complex synthetic gene circuits.

Despite the remarkable progress in synthetic biology, one of the most persistent challenges is the dependence on synthetic chemical inducers such as IPTG, anhydrotetracycline, or arabinose for controlling gene expression. While these inducers are highly effective in laboratory settings, they are impractical or undesirable in real-world applications, particularly in environmental monitoring, bioremediation, or in vivo diagnostics. To overcome these limitations, there is a growing interest in developing environmentally responsive regulatory systems, which can be activated or repressed by naturally occurring environmental cues such as pH, temperature, light, or the presence of specific pollutants. A wide range of such systems have already been engineered to detect heavy metals [20,21,22], organic pollutants like naphthalene [23], pesticides [24], and plastic-derived compounds [25,26,27]. Among these, benzoate stands out due to its widespread use as a preservative in food, beverages, and personal care products. It is released into the environment through industrial wastewater, landfill leachate, and agricultural runoff and can bioaccumulate in the food chain, ultimately reaching human consumers. Chronic exposure to benzoate has been linked to endocrine disruption and oxidative stress in aquatic organisms, raising concerns about its potential carcinogenic and mutagenic effects in mammals.

The central innovation of this work lies in the integration of multiple regulatory inputs (460 nm blue light, arabinose, and benzoate) into a unified, modular gene expression system capable of operating autonomously in complex, real-world environments. We first engineered a tripartite inducible system that responds to blue light, arabinose, and IPTG, allowing for precise, multi-layered control over gene expression. We then replaced the canonical Lac operator (LacO) with the benzoate-responsive operator (BenO) and substituted the LacI repressor with the benzoate-responsive transcription factor BenR from *Pseudomonas putida* KT2440. This modification transformed the system into a repression-based biosensor, in which benzoate acts as a negative regulator that reduces reporter gene expression in a concentration-dependent manner, making it particularly suitable for environmental sensing applications where pollutant detection relies on signal decrease. The system is designed to be highly modular, as individual components such as RBS variants, promoter architectures, and split T7RNAP variants can be easily swapped or optimized without disrupting the overall circuit design. This modularity enables rapid adaptation for detecting other small molecules by simply replacing the BenR/BenO module. However, current limitations include host dependence on engineered *E. coli* and the need for further optimization of dynamic range under non-laboratory conditions.

## 2. Results

### 2.1. Design of Light-Induced Gene Expression System

We engineered a blue-light-induced gene expression system by leveraging the light-sensitive domains nMag and pMag, derived from the fungal *Neurospora crassa* [28]. These domains have been extensively characterized and optimized for high dynamic range, rapid kinetics, and low dark-state activity. Notably, Kawano et al. systematically engineered and evaluated multiple variants of these photoswitches, identifying key mutations that enhance dimerization efficiency and reduce background binding in the absence of light [29]. To enable light-induced reconstitution of transcriptional activity, we split T7RNAP at its flexible loop region between amino acid residues 301 and 302 and fused the N-terminal fragment to nMag and the C-terminal fragment to pMag, via flexible peptide linkers [17]. Both fusion constructs were placed under the control of the ParaBAD, ensuring tightly regulated, inducible expression upon addition of L-arabinose. Each construct was followed by a transcriptional terminator to prevent readthrough and ensure proper termination of mRNA synthesis.

To monitor transcriptional output, we constructed a reporter plasmid in which the native promoter was replaced with a T7 promoter [30]. Downstream of the T7 promoter, we inserted a ribosome-binding site (RBS) and a multiple cloning site (MCS), allowing flexible insertion of reporter genes, such as fluorescent proteins or other functional genes. A transcriptional terminator was appended after the gene of interest to ensure precise termination and avoid aberrant transcription.

**Figure 1:**
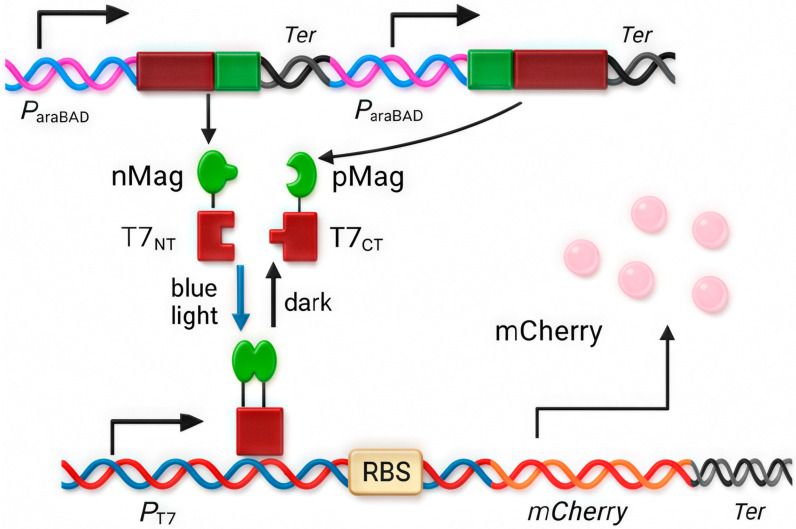
Schematic of the blue-light-inducible gene expression system. Blue light induces dimerization of nMag and pMag, leading to reassembly of split T7 RNA polymerase (T7NT and T7CT) into an active enzyme. The reconstituted T7RNAP drives transcription of the *mCherry* reporter gene from the T7 promoter (PT7), with translation efficiency modulated by the RBS. Transcription terminates at the Ter site. In the dark, the system remains inactive, resulting in no *mCherry* expression.

Upon addition of L-arabinose, the split T7RNAP fragments are expressed. Subsequent exposure to 460 nm blue light triggers a conformational change in nMag and pMag, inducing their rapid and reversible dimerization. This brings the two T7RNAP fragments into spatial proximity, reconstituting a functional RNA polymerase capable of binding the T7 promoter and initiating transcription of the downstream gene. Thus, gene expression is co-regulated by both arabinose and blue light, enabling precise, spatiotemporal control (Figure 1).

Importantly, nMag and pMag exhibit high orthogonality, showing negligible cross-reactivity with endogenous cellular proteins, and they dissociate rapidly upon light withdrawal, allowing dynamic, reversible regulation [31,32]. This system provides a robust, modular platform for optogenetic control of gene expression in bacterial systems, with potential applications in synthetic biology, metabolic engineering, and dynamic pathway regulation.

**Figure 2:**
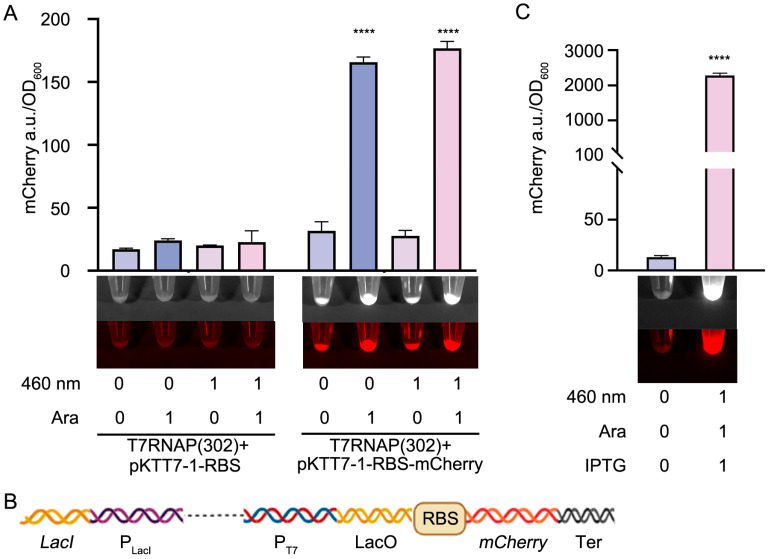
Characterization of gene expression in the blue-light-inducible system in *E. coli* K12. (**A**) *mCherry* expression under combinatorial induction with arabinose (Ara) and 460 nm blue light. Fluorescence intensity was quantified from liquid cultures and visualized by red fluorescence (inset). ****: *p* < 0.0001 (one-way ANOVA). (**B**) Schematic of the engineered plasmid incorporating the Lac operator (LacO) and LacI repressor for tighter transcriptional control. (**C**) Enhanced *mCherry* expression in the optimized system upon induction. ****: *p* < 0.0001 (unpaired *t*-test). “0”: no inducer/light; “1”: with inducer/light.

However, quantitative evaluation of fluorescence output revealed substantial basal leakage expression in the presence of arabinose alone without blue light irradiation (Figure 2A). Notably, this leakage level approached the expression intensity observed under dual induction (Ara + 460 nm blue light). Specifically, expression under dual induction was 5.6-fold higher than under the “dual-off” condition (no arabinose, no blue light). Even in the complete absence of both arabinose and blue light (“dual-off” condition), a low but detectable level of fluorescence was still observed, indicating non-specific background transcription. In contrast, when blue light was applied alone (without arabinose), leakage expression was significantly reduced, though it remained higher than the “dual-off” condition, suggesting that arabinose provides more precise control over the system’s “on/off” state [33]. The persistent leakage under blue light alone may stem from ambient environmental light, which often contains 460 nm components that inadvertently activate the nMag/pMag dimerization.

To address these limitations, we engineered a refined reporter plasmid by inserting a Lac operator (LacO) downstream of the T7 promoter and co-expressing a LacI repressor under the control of the PlacI promoter on the same plasmid (Figure 2B). This redesigned system requires tripartite induction: arabinose to express the split T7RNAP fragments, blue light to trigger nMag/pMag dimerization and polymerase reconstitution, and IPTG to relieve LacI-mediated repression at the LacO site. This triple-layered regulation dramatically enhances both specificity and dynamic range.

We then quantified *mCherry* expression levels under induced versus non-induced conditions (Figure 2C). Strikingly, full induction (Ara + blue light + IPTG) resulted in a 175-fold increase in fluorescence output compared to the uninduced state (all three inputs absent). The inset fluorescence images in Figure 2C visually corroborate this result: induced cultures exhibit intense red fluorescence, while non-induced cultures show negligible signal, confirming the system’s high signal-to-noise ratio and precise spatial-temporal control. The full induction matrix demonstrates that maximal expression is achieved only under full induction (Ara + blue light + IPTG), while all other conditions, including single or dual inductions, exhibit significantly lower output, validating the system’s orthogonality and tight repression (Figure S1).

In summary, the integration of the LacI/LacO repression module effectively suppresses background leakage while boosting inducible expression by 175-fold. This optimized system offers a powerful, high-fidelity platform for optogenetic control of gene expression in *E. coli*, with broad applicability in synthetic biology, metabolic engineering, and dynamic circuit design.

### 2.2. Effect of RBS on System Expression

**Figure 3:**
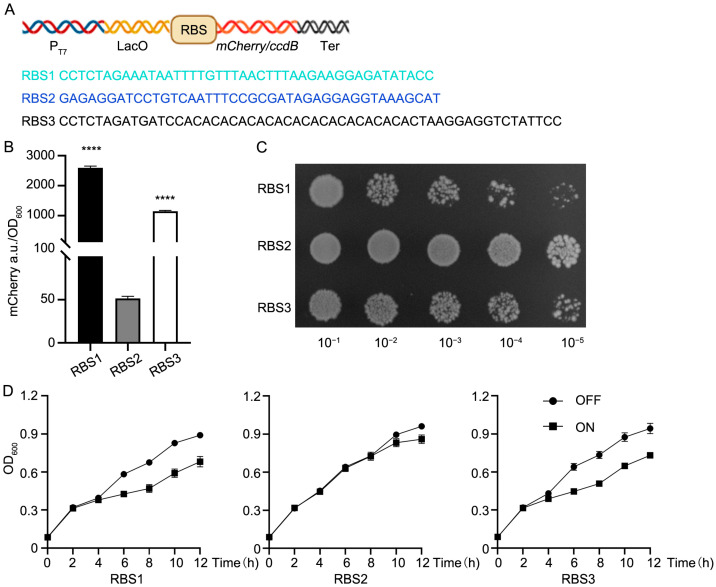
Impact of RBS variants on gene expression, bactericidal activity, and bacterial growth in *E. coli* K12. (**A**) DNA sequences of the three RBS variants (RBS1, RBS2, RBS3) inserted upstream of the *mCherry* reporter gene under the T7 promoter. (**B**) Quantification of *mCherry* expression levels under induced (ON) and uninduced (OFF) conditions (****: *p* < 0.0001, unpaired *t*-test). (**C**) Spot assay to assess bactericidal activity of each RBS variant. Serial 10-fold dilutions of bacterial cultures were spotted on agar plates after 12 h induction. Reduced colony formation indicates stronger bactericidal effect. (**D**) Growth curves of *E. coli* K12 harboring each RBS variant under induced (ON, black squares) and uninduced (OFF, black circles) conditions. Induced: full induction with L-arabinose, 460 nm blue light, and IPTG. All conditions include all three inducers unless otherwise noted.

The ribosome binding site (RBS) plays a pivotal role in modulating translation initiation efficiency, and even minor sequence variations among RBSs can lead to substantial differences in protein expression level [34,35]. To systematically evaluate the impact of RBS design on the output of our optogenetic system, we replaced the RBS sequence in the reporter plasmid with three distinct variants, RBS1, RBS2, and RBS3, as illustrated in Figure 3A. The RBS sequences used were selected from established, literature-based standardized designs, with their translation initiation rates (TIRs) computationally predicted. However, in our experimental system, RBS1 exhibited the highest expression efficiency, contrary to the prediction that RBS3 (TIR = 2.15) would be optimal (Table S3). We then quantified mCherry fluorescence expression under each condition. Our results revealed that the RBS2-driven system exhibited the lowest expression level, approaching the basal leakage observed under uninduced conditions. In contrast, expression from the RBS3 system was approximately 60% lower than that of RBS1 (Figure 3B), highlighting the sensitivity of translation efficiency to RBS sequence. The fluorescence imaging in Figure S2A did not visually resolve a clear intensity difference between RBS1 and RBS3, yet the overall fluorescence distribution aligned with the quantitative trend: RBS1 exhibited the strongest signal, RBS2 the weakest, and RBS3 an intermediate level.

To assess the physiological-level functional impact of RBS variation on system output, we cloned the toxin gene *ccdB* into the reporter plasmid and performed spot assays on agar plates supplemented with arabinose and IPTG, under continuous 460 nm blue light irradiation. As shown in Figure 3C, colony formation decreased progressively with increasing dilution, indicating a dose-dependent bactericidal effect upon system activation. As a control, cultures grown in the absence of inducers and under dark conditions formed normal, dense colonies across all dilutions (Figure S2B), confirming that system activation strictly depends on the tripartite induction regimen.

To further dissect the temporal dynamics of toxin-mediated killing, we monitored bacterial growth kinetics for each RBS variant following induction. Consistent with the spot assay results, RBS1 and RBS3 systems exhibited significant growth inhibition within 2 h of induction, reflecting rapid toxin accumulation and activity. In contrast, RBS2 showed minimal growth suppression until 8 h post-induction, indicating severely delayed and inefficient translation of *ccdB* (Figure 3D). This demonstrates that RBS selection not only modulates expression magnitude but also governs the kinetics of functional output.

In summary, this study systematically characterizes how RBS engineering tunes both the amplitude and temporal dynamics of gene expression in our optogenetic system. These findings provide a practical framework for selecting optimal RBS sequences to match desired expression levels in diverse applications, ranging from low-background biosensing to high-output toxin delivery, thereby significantly expanding the system’s programmability and functional versatility.

### 2.3. Modulation of Expression Levels by Tandem T7 Promoters

**Figure 4:**
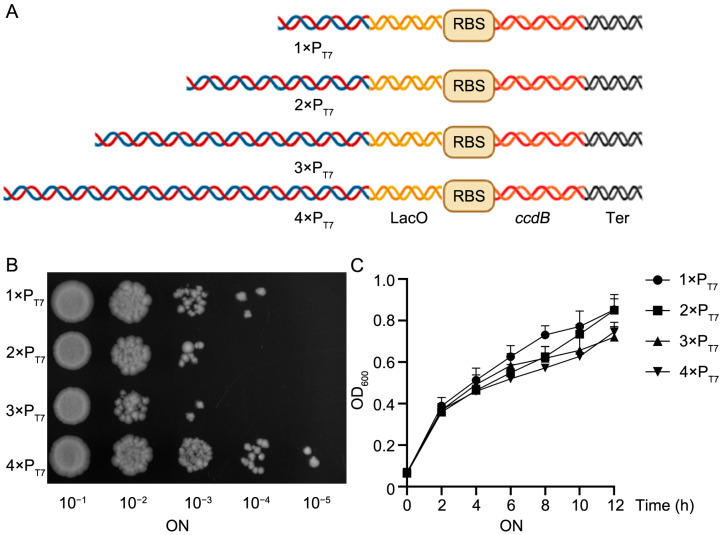
Impact of T7 promoter copy number on gene expression and cell growth. (**A**). Schematic diagrams of DNA constructs containing 1 to 4 tandem copies of the T7 promoter. (**B**). Spot assay of *E. coli* strains harboring different P_T7_ copy numbers. Reduced colony formation with higher promoter copy number indicates enhanced *ccdB* toxicity, reflecting elevated system expression. (**C**). Growth curves of strains with varying P_T7_ copies. ON: induced.

Promoters are core regulatory elements that initiate transcription, and their copy number can profoundly influence transcriptional output and downstream protein expression. Previous studies have shown that tandem arrangement of three tac promoters significantly enhances the biosynthesis yield of 3-hydroxypropionic acid, suggesting that multi-promoter architectures can effectively amplify transcriptional output [36]. To investigate whether T7 promoters exhibit similar behavior upon tandem duplication, we constructed a series of reporter plasmids harboring one, two, three, or four tandem copies of the T7 promoter (Figure 4A), with no intervening spacer sequences between promoters to avoid introducing unintended regulatory elements. The RBS1 variant was selected for these constructs due to its superior translation efficiency demonstrated in the preceding characterization. The toxin gene *ccdB* was subsequently cloned into each plasmid, and the bactericidal efficacy of each system was evaluated via spot assay under inducing conditions.

As shown in Figure 4B, when cultures were plated on agar supplemented with arabinose and IPTG and exposed to 460 nm blue light, colony formation decreased markedly with increasing dilution for strains carrying two or three tandem T7 promoters, indicating robust toxin expression and potent bactericidal activity. In contrast, the system with four tandem T7 promoters showed significantly reduced killing efficiency: colonies remained abundant even at 10^−5^ dilution, likely due to transcriptional interference, RNA polymerase congestion, or mRNA instability, all of which diminish functional output. As a control, under uninduced conditions (no arabinose, no IPTG, no blue light), all systems formed normal, dense colonies across all dilutions (Figure S3B), confirming that bactericidal activity is strictly dependent on the tripartite induction regimen.

To further dissect the temporal dynamics of toxin-mediated killing, we monitored bacterial growth kinetics for each promoter variant following induction (Figure 4C). The growth curves aligned closely with the spot assay results: systems with two or three T7 promoters exhibited observable growth inhibition, whereas the four-promoter system showed minimal growth suppression, with kinetics nearly identical to the uninduced control. These findings collectively suggest that three tandem T7 promoters represent the optimal configuration for balancing transcriptional output and system fidelity in this optogenetic platform.

Notably, the *mCherry* expression data in Figure S3A reveal that triplication of the T7 promoter (3 × T7) yields the highest expression level, surpassing both single and quadruple copies. This concordance between direct fluorescence measurement and phenotypic output strongly validates the use of bacterial growth as a reliable, high-throughput proxy for gene expression in our system.

In summary, this study provides the first systematic evaluation of T7 promoter copy number on gene expression and functional output in a light-inducible system. Our results reveal a non-linear relationship between promoter dosage and system performance, with three tandem T7 promoters achieving the highest functional efficiency. These findings offer valuable design principles for tuning transcriptional output in synthetic biology applications, enabling precise control over gene expression levels for diverse biotechnological purposes, ranging from high-yield metabolite production to dynamic toxin delivery systems.

### 2.4. Effect of T7RNAP Split Site on System Expression Efficiency

**Figure 5:**
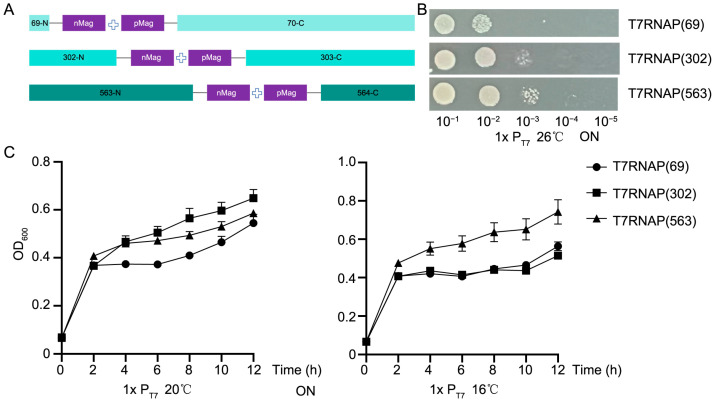
Impact of T7RNAP split sites on system expression efficiency. (**A**) Schematic representation of T7RNAP split at positions 69, 302, and 563. Promoter: ParaBAD (arabinose-inducible); reporter gene: *mCherry*. (**B**) Bactericidal activity of systems with different split sites, evaluated by spot assays (dilution series: 10^−1^ to 10^−5^). (**C**) Growth kinetics (OD_600_ over time) of systems with different split sites under induction at 20 °C (left) and 16 °C (right), respectively. All experiments utilize 1 × T7. All inducers (Ara, IPTG, and 460 nm blue light) were added during the experiment. ON: induced.

T7RNAP contains multiple flexible loop regions, and previous studies have demonstrated that the efficiency of functional domain fusion varies depending on the split site [13,37]. In our prior work, we consistently selected residue 302 as the split site to enable reversible reconstitution of T7RNAP in our optogenetic system. To explore whether alternative flexible loop regions could serve as more effective or environmentally adaptive split sites, we engineered additional constructs by splitting T7RNAP at residues 69 and 563 (Figure 5A). Following the same design principle as the 302-split variant, the N-terminal fragment was fused to nMag and the C-terminal fragment to pMag, and both were cloned downstream of the arabinose-inducible promoter. To evaluate functional output, particularly the bactericidal activity conferred by the toxin gene *ccdB*, all systems were co-expressed with a reporter plasmid driven by a single T7 promoter.

The observed temperature-dependent performance aligns with the known maturation kinetics of Magnet domains, which peak near 28 °C in *E. coli* [9]. Our data show that system efficiency increases as temperature approaches this window, highlighting the importance of thermal context in optogenetic circuit design. First, we performed spot assays at 26 °C (Figure 5B). The results revealed that the T7RNAP (69) system exhibited the strongest bactericidal activity under induction, with colony formation markedly reduced at higher dilutions. The T7RNAP (302) system showed intermediate efficacy, while the T7RNAP (563) system, although functional, displayed significantly lower killing efficiency compared to the other two. As a control, all systems exhibited normal colony formation under uninduced conditions (no arabinose, no IPTG, no blue light), confirming that bactericidal activity is strictly dependent on the tripartite induction regimen (Figure S4A).

To evaluate the system’s dynamic response, we performed additional growth curve experiments at 26 °C, which confirmed the plate-based observations: the system remains functional at 26 °C, but with reduced expression efficiency (Figure S4C).

To further dissect the temperature-dependent dynamics of toxin-mediated killing, we monitored bacterial growth kinetics at 20 °C and 16 °C (Figure 5C). At 20 °C, the T7RNAP (69) system again demonstrated the most potent growth inhibition. The T7RNAP (302) system followed closely, while T7RNAP (563) showed the weakest inhibition. Strikingly, when the temperature was lowered to 16 °C, the T7RNAP (302) system exhibited markedly enhanced bactericidal efficiency, surpassing even the T7RNAP (69). This suggests that residue 302 may confer superior structural stability or polymerase reconstitution kinetics under cold stress. Notably, no significant differences in growth kinetics were observed among any of the systems under uninduced conditions across all temperatures (Figure S4B), further validating the system’s tight regulatory control.

To evaluate how multiple optimization strategies interact within the same genetic circuit, we systematically analyzed the combined effects of T7 promoter copy number and RNAP split site configuration. Our data demonstrate that these modifications exert an additive effect on transcriptional output. Specifically, when the optimized split site was combined with an increasing number of tandem T7 promoters, gene expression was further amplified compared to systems containing only a single promoter or a single optimization parameter. This additive relationship indicates that promoter dosage and polymerase reconstitution efficiency operate through partially independent mechanisms, allowing their regulatory benefits to accumulate. These findings confirm the modular scalability of the platform and demonstrate that integrating multiple optimized elements significantly enhances the overall dynamic range and robustness of the system. The complete comparative data are presented in Supplementary Figure S4D.

In summary, this study provides the first systematic evaluation of T7RNAP split site selection on the functional output of an optogenetic expression system. Our findings reveal a non-linear, temperature-dependent relationship between split site position and system performance: T7RNAP (69) is optimal at moderate temperatures (20–26 °C), while T7RNAP (302) outperforms others at low temperatures (16 °C). These results offer valuable engineering guidelines for tailoring optogenetic systems to environmental conditions, significantly expanding their applicability in synthetic biology, ranging from laboratory-scale control to field-deployable biosensors or temperature-responsive therapeutic systems.

### 2.5. Design and Characterization of a Benzoate-Responsive Genetic Circuit via Lac Operon Replacement

We aim to develop a genetically encoded regulatory system that can be deployed in real-world environmental settings. However, conventional laboratory inducers such as IPTG are absent in natural environments. Therefore, it is essential to engineer our system to respond to endogenous environmental cues and small molecules naturally present in ecosystems, rather than relying on synthetic chemical inducers. Inspired by prior studies demonstrating that salicylic acid can serve as an environmental signal to modulate synthetic gene circuits and output fluorescent reporter signals [38], we expanded our scope to investigate other environmentally relevant small molecules, particularly organic pollutants such as benzoate, which are widespread in contaminated soils and aquatic systems and are known to act as signaling molecules in certain bacterial species.

**Figure 6:**
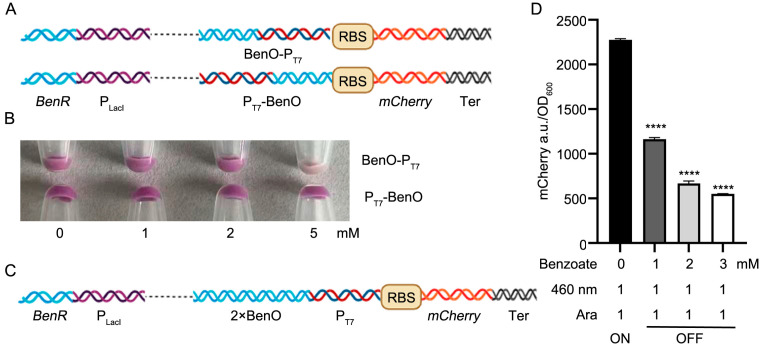
Construction and functional characterization of a benzoate-regulated gene expression system. (**A**). Schematic diagram of the DNA construct in which the lactose-operon-regulatory elements are replaced by benzoate-responsive elements. (**B**). Effect of BenO placement (upstream or downstream of the PT7 promoter) on system expression, monitored via mCherry fluorescence. (**C**). Schematic of the DNA construct containing tandem repeats of the BenO element. (**D**). Dose-dependent response of the system to increasing benzoate concentrations, quantified as normalized mCherry fluorescence. “no benzoate” = ON state (maximal expression), and “benzoate added” = OFF state (expression repressed). “1”: with Ara/light. All experiments utilize 1 × T7 and were performed with Ara and 460 nm blue light. ****: *p* < 0.0001 (one-way ANOVA).

To this end, we mined the genomes of benzoate-metabolizing bacteria (*Pseudomonas* KT2440) to identify key components of the native benzoate-responsive regulatory system: the transcriptional regulator BenR and its cognate operator sequence BenO [39,40]. The BenR/BenO module used in this study is not novel per se but is reported here for the first time integrated into a light-controlled split T7 RNAP system to create the first tri-input gene expression platform responsive to blue light, arabinose, and benzoate. The system functions through a classic negative regulation mechanism: in the presence of benzoate, the small molecule binds to the regulatory repressor protein, enabling it to attach to the operator sequence and inhibit downstream gene transcription. Conversely, when benzoate is absent, the repressor dissociates from the DNA, freeing the promoter region and allowing RNA polymerase to bind and drive efficient transcription (Figure S5). Leveraging these natural genetic parts, we performed a modular redesign of the classic Lac operon-based system. Specifically, we replaced the LacI repressor with BenR, removed the original LacO operator, and strategically inserted BenO sequences either upstream or downstream of the T7 promoter, which drives expression of the red fluorescent reporter *mCherry* in conjunction with T7RNAP (302). This configuration allows the system to respond to extracellular benzoate concentration via BenR-mediated repression (Figure 6A).

As shown in Figure 6B, when BenO is positioned upstream of the T7 promoter, the system exhibits a clear dose-dependent repression of *mCherry* expression with increasing benzoate concentration, indicating successful integration of the environmental signal into the transcriptional output. In contrast, when BenO is placed downstream of the promoter, no repression is observed, confirming that the spatial orientation of the operator relative to the promoter is critical for functional regulation. Both systems peaked at 1–2 mM benzoate, but expression declined sharply at 5 mM—2.7-fold in BenO-T7 and 2.2-fold in T7-BenO—relative to 0 mM (Figure S6), suggesting higher inducer sensitivity in BenO-T7. To further enhance system sensitivity, we engineered a tandem BenO array upstream of the T7 promoter (Figure 6C), with no intervening spacer sequences, to promote cooperative binding of BenR and thereby strengthen repression at lower inducer concentrations. Quantitative fluorescence measurements revealed that, at a benzoate concentration of 3 mM, the expression of *mCherry* was reduced by 4.5-fold compared to the uninduced control, demonstrating a significant improvement in dynamic range and sensitivity (Figure 6D).

This study successfully establishes a synthetic gene circuit responsive to environmental benzoate, offering a novel strategy for environmental monitoring using engineered microbes. The system is inducer-free, environmentally compatible, and modularly adaptable, making it a promising candidate for deployment in soil, wastewater, or bioremediation systems where real-time detection of organic pollutants is critical. Future work will focus on optimizing promoter strength, expanding the range of detectable pollutants, and integrating the system into robust chassis organisms for field applications.

## 3. Discussion

In this study, we engineered a multi-input, light-responsive gene expression system capable of precise transcriptional control under the combined regulation of arabinose, IPTG, and blue light. We systematically evaluated the impact of RBS variants on protein output, revealing up to a 50-fold difference in expression between RBS1 and RBS2. Through promoter engineering, we identified that tandem T7 promoters, which consist of three copies arranged in series, maximized both transcriptional output and bactericidal efficacy. Furthermore, we replaced the Lac operon with a benzoate-responsive module derived from *Pseudomonas putida* KT2440, thereby constructing a novel tripartite inducible system responsive to benzoate, arabinose, and blue light. Crucially, unlike IPTG, benzoate acts as a repressor in our system: when two BenO operator sites were placed upstream of the T7 promoter, 3 mM benzoate induced a 4.5-fold reduction in reporter gene expression. Collectively, these findings establish a modular, tunable, and environmentally responsive gene circuit with significant potential for applications in biosensing, bioremediation, and smart microbial therapeutics.

The observed 50-fold variation in fluorescent protein expression between RBS1 and RBS2 underscores the pivotal role of translational regulation in synthetic gene circuits. RBS directly governs ribosome binding affinity and translation initiation efficiency, making it a powerful “knob” for modulating protein output without altering transcriptional machinery. This finding aligns with prior work, which demonstrated that RBS libraries can generate expression differences with significant magnitude in *Synechocystis* sp. PCC 6803 [41]. In our design, we deliberately selected RBS variants that minimize secondary RNA structure formation, thereby enhancing the reproducibility and predictability of expression levels, which is a critical consideration for robust circuit design [42]. The ability to precisely tune translation efficiency is particularly valuable in applications requiring stoichiometric control of multi-subunit complexes or threshold-dependent logic gates (e.g., in biosensors or therapeutic circuits). Moreover, our RBS library provides a flexible engineering toolkit: weaker RBSs can be paired with stronger promoters to mitigate metabolic burden, while stronger RBSs can be coupled with weaker promoters to achieve intermediate expression levels, which enables context-specific optimization for diverse host environments or functional requirements.

We observed that three tandem T7 promoters yielded the highest gene expression and bactericidal efficacy, which maintained low basal leakage. Although T7 promoters are widely used for their orthogonality and high transcriptional strength, our initial experiments with single T7 promoters revealed suboptimal expression levels, prompting us to explore the use of tandem promoters. Contrary to the intuitive assumption that more promoters equal higher output, we found that gene expression did not scale linearly with promoter copy number. Instead, expression peaked at three tandem copies and declined with four or more, consistent with findings that showed a plateau in expression after four tandem promoters in *E. coli* [43]. This non-linear behavior likely stems from steric hindrance or reduced T7 RNA polymerase (T7RNAP) binding efficiency when promoter density exceeds a critical threshold. Importantly, no significant increase in basal leakage was observed with tandem promoters, indicating that our design preserves tight transcriptional control, which is a prerequisite for applications in biosensors, logic gates, or therapeutic gene circuits where unintended expression must be minimized. The graded survival observed under *ccdB* induction reflects dose-dependent toxicity rather than system insensitivity or mutational escape, consistent with established toxin-based circuit behavior. Short assay durations (≤24 h) and consistent replicates further minimize escape risk.

Previous studies have shown that T7RNAP can be functionally split at multiple positions without compromising activity upon reconstitution. In our work, we tested multiple split sites and discovered that the optimal split position varied with temperature (Figure 5). Although 28 °C optimizes Magnet maturation, we screened 16–26 °C to assess thermal robustness and identify application-relevant operational windows, as environmental temperatures in biosensing/biocontrol contexts often fall below 28 °C. This range also allows evaluation of temperature-dependent trade-offs between photosensor functionality, promoter kinetics, and expression leakiness. This temperature-dependent fusion efficiency suggests that different split variants can be selected to optimize performance under distinct environmental conditions. This finding significantly broadens the environmental applicability of our system, making it suitable for deployment in diverse ecological niches, including soil, which is typically cooler and more variable, and wastewater, which is generally warmer and more stable, without requiring system redesign. It also opens new avenues for environmentally responsive synthetic biology, where gene expression can be tuned not only by chemical inducers but also by physical parameters such as temperature.

The most innovative aspect of this work is the integration of a benzoate-responsive regulatory module into our blue-light-controlled system, resulting in a novel tripartite control system responsive to benzoate, arabinose, and blue light. Unlike the system constructed by Uchiyama et al. [44], where inducers activate expression, benzoate functions as a repressor in our circuit. Specifically, we replaced the Lac operator (LacO) with the BenO operator and substituted LacI with the benzoate-responsive transcription factor BenR from *Pseudomonas putida* KT2440. In the absence of benzoate, BenR does not bind BenO, allowing T7RNAP to access the promoter and drive reporter gene expression. Upon addition of benzoate, BenR undergoes a conformational change, enabling it to bind BenO with high affinity and sterically block T7RNAP binding, thereby repressing transcription [40,45]. When two BenO sites were placed upstream of the T7 promoter, we observed a 4.5-fold reduction in reporter expression at 3 mM benzoate, which is a repression level comparable to that reported for salicylate-responsive systems. Notably, this contrasts with the work of Uchiyama et al., who engineered a benzoate-inducible system in which gene expression increased with benzoate concentration, which highlights the versatility of BenR/BenO as a regulatory module that can be configured for either activation or repression, depending on promoter architecture and operator placement.

Notably, recent work by Baldanta and Rodrigo also demonstrated that layered tripartite control (aTc, light, and arabinose) effectively minimizes basal leakage in OptoT7 systems [46]. While our approach utilizes a benzoate-responsive BenR/BenO module rather than a tet-riboregulator, both studies independently validate that adding orthogonal chemical layers significantly improves transcriptional precision in split T7 architectures. Compared to traditional inducible systems (e.g., lac, tet, ara), our benzoate-responsive circuit offers several key advantages for environmental applications. First, benzoate, as a naturally occurring environmental pollutant, directly represses the system, enabling a self-contained “sense-and-report” mechanism. Second, its modular design allows for easy swapping of regulatory elements, such as replacing BenR/BenO with other transcription-factor–operator pairs to detect different pollutants (e.g., phthalates, phenols, or heavy metals). However, several limitations must be addressed. First, our system has so far been validated only in *E. coli*; its performance in other chassis organisms (e.g., *Pseudomonas*, *Bacillus*) remains untested. Second, we observed that high benzoate concentrations (>3 mM) inhibit bacterial growth, suggesting potential metabolic burden or toxicity that may limit system utility in highly contaminated environments. Future work will focus on (1) enhancing repression sensitivity through promoter engineering or auxiliary regulatory elements; (2) testing system robustness in diverse microbial hosts and under environmental stressors (pH, temperature, nutrient limitation); and (3) integrating the system with signal amplification or wireless readout modules for real-time, field-deployable biosensing. Recent advances have significantly refined the photophysical properties of Magnets photosensors. For instance, Baumschlager et al. demonstrated that directed evolution can independently tune light sensitivity, activation efficiency, and dose–response kinetics, providing a customizable toolkit for single-input optogenetic circuits [47]. While these studies excel at component-level optimization, our work addresses a complementary challenge: integrating light-responsive elements into multi-input genetic architectures that function robustly under environmentally relevant conditions. Together, these efforts highlight the progressive maturation of optogenetic tools from isolated part characterization to system-level application.

In conclusion, we have successfully constructed a multi-input, light-responsive gene expression system that integrates RBS tuning, tandem promoter engineering, split T7RNAP design, and benzoate-responsive repression into a single, modular platform. Key findings include the following: (i) RBS variants enable up to 50-fold expression modulation; (ii) three tandem T7 promoters maximize output without increasing leakage; (iii) split T7RNAP variants allow temperature-adaptive performance; and (iv) BenR/BenO integration enables 4.5-fold repression at 3 mM benzoate. This system represents a significant step toward practical environmental synthetic biology, where engineered microbes can autonomously sense, respond to, and report on environmental pollutants. Its modularity, tunability, and environmental compatibility make it a promising candidate for applications in biosensing, bioremediation, and smart microbial therapeutics. Future iterations will focus on enhancing sensitivity, expanding host range, and integrating with real-time monitoring technologies, which will ultimately contribute to a more sustainable, responsive, and intelligent approach to environmental management. In the future, such systems could be integrated with microfluidic devices, wireless communication modules, or cell-free expression systems to enable real-time, remote monitoring of environmental health, thereby bringing synthetic biology out of the lab and into the field.

## 4. Materials and Methods

### 4.1. Strains and Media

*Escherichia coli* TG1 was used for plasmid construction and cloning, while *Escherichia coli* K12 strains were employed for phenotypic assays. All strains were routinely cultured in Luria–Bertani (LB) medium at 37 °C with shaking. When required, antibiotics were added to the medium: kanamycin (50 μg/mL) for selection of KanR-containing plasmids, and ampicillin (100 μg/mL) for AmpR-containing plasmids. For induction of gene expression, 0.2% L-arabinose or 1 mM IPTG was added to the culture medium as appropriate. Unless otherwise specified, all routine experiments (including Figure 2 and Figure 3) were conducted at 26 °C. For growth curve assays, cultures were shifted from 37 °C to 26 °C upon inducer addition, with controls maintained under identical conditions. All the strains used were obtained from our laboratory, and the reagents were purchased from Solarbio Science & Technology Co., Ltd. (Beijing, China).

### 4.2. Plasmid Construction

A second open reading frame (ORF) under the control of the arabinose-inducible promoter (ParaBAD) was inserted into the pBAD22a backbone, along with engineered restriction sites to facilitate subsequent cloning. The *mCherry* gene was inserted into both multiple cloning sites (MCSs) to validate expression efficiency, ensuring that the system remains functional when *mCherry* is subsequently replaced with the N- and C-terminal fragments of T7RNAP. T7RNAP was split at different positions and fused to the light-sensitive domains nMag or pMag via flexible linkers. These constructs were cloned into the two MCSs to enable independent expression of the N- and C-terminal fragments under arabinose induction, followed by light-dependent reassembly into functional T7RNAP upon exposure to 460 nm blue light. The reporter plasmid was constructed by replacing the native promoter in pKT100 with a T7 promoter and introducing a new multiple cloning site (MCS) to facilitate insertion of reporter genes for monitoring T7RNAP activity. The lacI gene and other Lac operon elements were derived from pET28a. The ribosome-binding site (RBS) was incorporated into the primers and replaced via PCR amplification. Additionally, tandem promoter sequences were introduced via primer design to enable promoter duplication. All plasmid constructions were performed using seamless cloning techniques to ensure precise, scarless ligation without extraneous nucleotides. The constructed split-T7RNAP expression plasmid and the T7-promoter-driven reporter plasmid were co-electroporated into *E. coli* K12 for functional characterization. All plasmids used in this study are listed in Supplementary Table S1, and all primers are listed in Supplementary Table S2.

All system components are expressed from two distinct plasmids. The expression plasmid carries the N-terminal T7 RNAP fragment fused to nMag and the C-terminal fragment fused to pMag, both under the control of the arabinose-inducible ParaBAD promoter. The reporter plasmid contains the T7 promoter driving expression of the *mCherry* reporter gene, followed by a Lac operator (LacO) sequence and the *LacI* repressor gene under the control of the PlacI promoter. This dual-plasmid design enables independent regulation of polymerase expression and reporter output while allowing precise suppression of basal leakage via LacI–LacO repression.

Except for Figure 3, where different RBS variants are tested as the sole variable, all other experiments in the manuscript use RBS1 as the standard ribosome binding site.

### 4.3. Growth Curve Assay

Activated bacterial cultures were adjusted to OD600 = 1.0 and inoculated at 1% (*v*/*v*) into fresh LB medium. After 2 h of incubation at 37 °C with shaking, inducers (0.2% L-arabinose or 1 mM IPTG) were added, and 460 nm blue light illumination was simultaneously initiated. The culture temperature was then reduced to 26 °C to optimize the balance between protein expression and cell growth under induction conditions. OD600 was measured every 2 h for up to 12 h, and growth curves were plotted to evaluate bacterial growth kinetics under induced conditions.

### 4.4. Spot Assay

Activated bacterial cultures were adjusted to OD_600_ = 1.0 and serially diluted in sterile water (e.g., 10^−1^ to 10^−5^). After thorough mixing, 2 μL of each dilution was spotted onto LB agar plates containing inducers (0.2% L-arabinose or 1 mM IPTG). Plates were incubated under 460 nm blue light at 26 °C for 12 h to assess strain growth and induction responsiveness across dilution series.

### 4.5. Fluorescence Intensity Measurement

Activated bacterial cultures were adjusted to OD_600_ = 1.0 and inoculated at 1% (*v*/*v*) into fresh LB medium containing inducers (0.2% L-arabinose or 1 mM IPTG). Cultures were incubated at 26 °C with shaking (220 rpm) under continuous 460 nm blue light illumination for 12 h. After incubation, the culture supernatant was directly transferred to a black-bottom, clear-side 96-well microplate. Fluorescence intensity was measured using a SpectraMax M2 multi-mode microplate reader (Molecular Devices, San Jose, CA, USA) with excitation at 587 nm and emission at 610 nm for mCherry. OD_600_ was simultaneously measured for normalization.

### 4.6. Data Analysis

All experiments were performed in triplicate (*n* = 3), and data are presented as mean ± SEM. Statistical analyses were conducted using GraphPad Prism 8. Group comparisons were performed using one-way ANOVA; if significant differences were detected (*p* < 0.05), Tukey’s multiple comparisons test was applied for post hoc analysis. For two-group comparisons, an unpaired *t*-test was used. All statistical graphs were generated using GraphPad Prism 8.

## Data Availability

All the data supporting this finding in our study were included in this article and Supplementary Information files. Further inquiries can be made to the corresponding authors if necessary.

## Supplementary Materials

The following supporting information can be downloaded at: https://www.mdpi.com/article/10.3390/molecules31122032/s1, Figure S1, Full set of experimental controls for the tripartite inducible system; Figure S2, Evaluation of *mCherry* expression driven by different RBS sequences; Figure S3, Correlation between *mCherry* expression and *ccdB*-mediated growth inhibition under varying T7 promoter copy numbers; Figure S4, Effect of T7RNAP cleavage site position on system expression; Figure S5, Schematic representation of the benzoate-negative regulatory module; Figure S6, Dose-dependent response of two engineered benzoate-responsive systems, BenO-T7 (BenO operator upstream of T7 promoter) and T7-BenO (T7 promoter upstream of BenO operator), to increasing concentrations of benzoic acid (0–5 mM); Table S1, Plasmids used in this study; Table S2, Primers used in this study; Table S3. Genetic parts used in this study.

## Abbreviations

The following abbreviations are used in this manuscript:

T7RNAP	T7 RNA polymerase
IPTG	Isopropyl β-D-1-thiogalactopyranoside
LB	Luria–Bertani
RBS	Ribosome Binding Site
MCS	multiple cloning site

## References

[1] Lee J.W., Chan C.T.Y., Slomovic S., Collins J.J. (2018). Next-generation biocontainment systems for engineered organisms. Nat. Chem. Biol..

[2] Li W., Zhou Z., Li X., Ma L., Guan Q., Zheng G., Liang H., Yan Y., Shen X., Wang J. (2022). Biosynthesis of plant hemostatic dencichine in *Escherichia coli*. Nat. Commun..

[3] Wang D., Wang J., Shi Y., Li R., Fan F., Huang Y., Li W., Chen N., Huang L., Dai Z. (2020). Elucidation of the complete biosynthetic pathway of the main triterpene glycosylation products of *Panax notoginseng* using a synthetic biology platform. Metab. Eng..

[4] Shi Y., Wang D., Li R., Huang L., Dai Z., Zhang X. (2021). Engineering yeast subcellular compartments for increased production of the lipophilic natural products ginsenosides. Metab. Eng..

[5] Yan X., Fan Y., Wei W., Wang P., Liu Q., Wei Y., Zhang L., Zhao G., Yue J., Zhou Z. (2014). Production of bioactive ginsenoside compound K in metabolically engineered yeast. Cell Res..

[6] Zhang J., Hansen L.G., Gudich O., Viehrig K., Lassen L.M.M., Schrübbers L., Adhikari K.B., Rubaszka P., Carrasquer-Alvarez E., Chen L. (2022). A microbial supply chain for production of the anti-cancer drug vinblastine. Nature.

[7] Ng I.S., Keskin B.B., Tan S.I. (2020). A Critical Review of Genome Editing and Synthetic Biology Applications in Metabolic Engineering of *Microalgae* and *Cyanobacteria*. Biotechnol. J..

[8] Guo Y., Chen X., Gong P., Long H., Wang J., Yang W., Yao W. (2024). *Siraitia grosvenorii* As a Homologue of Food and Medicine: A Review of Biological Activity, Mechanisms of Action, Synthetic Biology, and Applications in Future Food. J. Agric. Food Chem..

[9] Benedetti L., Marvin J.S., Falahati H., Guillén-Samander A., Looger L.L., De Camilli P. (2020). Optimized Vivid-derived Magnets photodimerizers for subcellular optogenetics in mammalian cells. eLife.

[10] Guo S., Mao B., Tang X., Zhang Q., Zhao J., Chen W., Cui S. (2026). Autoinducer 2 as a universal language in microbial consortia: Decoding molecular mechanisms, ecological impacts, and application. Gut Microbes.

[11] Hallett R.A., Zimmerman S.P., Yumerefendi H., Bear J.E., Kuhlman B. (2015). Correlating in Vitro and in Vivo Activities of Light-Inducible Dimers: A Cellular Optogenetics Guide. ACS Synth. Biol..

[12] Chen X., Liu R., Ma Z., Xu X., Zhang H., Xu J., Ouyang Q., Yang Y. (2016). An extraordinary stringent and sensitive light-switchable gene expression system for bacterial cells. Cell Res..

[13] Baumschlager A., Aoki S.K., Khammash M. (2017). Dynamic Blue Light-Inducible T7 RNA Polymerases (Opto-T7RNAPs) for Precise Spatiotemporal Gene Expression Control. ACS Synth. Biol..

[14] Ohlendorf R., Vidavski R.R., Eldar A., Moffat K., Möglich A. (2012). From Dusk till Dawn: One-Plasmid Systems for Light-Regulated Gene Expression. J. Mol. Biol..

[15] Romano E., Baumschlager A., Akmeriç E.B., Palanisamy N., Houmani M., Schmidt G., Öztürk M.A., Ernst L., Khammash M., Di Ventura B. (2021). Engineering AraC to make it responsive to light instead of arabinose. Nat. Chem. Biol..

[16] Zhang Q., Ren J., Wu S., Tan Y., Wang W., Feng C., Zhao L., Zhu Z. (2025). Plasmid-Free CRISPR/Cpf1 Genome Editing With In Vivo T7 RNA Polymerase-Transcribed CRISPR RNA From Short Double-Stranded DNA. Biotechnol. Bioeng..

[17] Han T., Chen Q., Liu H. (2016). Engineered Photoactivatable Genetic Switches Based on the Bacterium Phage T7 RNA Polymerase. ACS Synth. Biol..

[18] Chiurillo M.A., Ahmed M., González C., Rosón J.N., Das A., Lander N. (2026). Cloning-Free Genome Editing by CRISPR/T7RNAP/Cas9 in Trypanosoma cruzi. Methods Mol. Biol..

[19] McCutcheon S.R., Chiu K.L., Lewis D.D., Tan C. (2017). CRISPR-Cas Expands Dynamic Range of Gene Expression From T7RNAP Promoters. Biotechnol. J..

[20] Guo Y., Hai Y. (2021). Adaptive surface mesh remeshing based on a sphere packing method and a node insertion/deletion method. Appl. Math. Model..

[21] Husser C., Vuilleumier S., Ryckelynck M. (2022). FluorMango, an RNA-Based Fluorogenic Biosensor for the Direct and Specific Detection of Fluoride. Small.

[22] Žunar B., Mosrin C., Bénédetti H., Vallée B. (2022). Re-engineering of CUP1 promoter and Cup2/Ace1 transactivator to convert Saccharomyces cerevisiae into a whole-cell eukaryotic biosensor capable of detecting 10 nM of bioavailable copper. Biosens. Bioelectron..

[23] Sun Y., Zhao X., Zhang D., Ding A., Chen C., Huang W.E., Zhang H. (2017). New naphthalene whole-cell bioreporter for measuring and assessing naphthalene in polycyclic aromatic hydrocarbons contaminated site. Chemosphere.

[24] Fan X., Zhao M., Wen H., Zhang Y., Zhang Y., Zhang J., Liu X. (2023). Enhancement degradation efficiency of pyrethroid-degrading esterase (Est816) through rational design and its application in bioremediation. Chemosphere.

[25] Zhang A., Hou Y., Wang Y., Wang Q., Shan X., Liu J. (2023). Highly efficient low-temperature biodegradation of polyethylene microplastics by using cold-active laccase cell-surface display system. Bioresour. Technol..

[26] Liu Y., Liu Z., Guo Z., Yan T., Jin C., Wu J. (2022). Enhancement of the degradation capacity of IsPETase for PET plastic degradation by protein engineering. Sci. Total Environ..

[27] Dai L., Qu Y., Huang J., Hu Y., Hu H., Li S., Chen C., Guo R. (2021). Enhancing PET hydrolytic enzyme activity by fusion of the cellulose—binding domain of cellobiohydrolase I from *Trichoderma reesei*. J. Biotechnol..

[28] Zoltowski B.D., Schwerdtfeger C., Widom J., Loros J.J., Bilwes A.M., Dunlap J.C., Crane B.R. (2007). Conformational switching in the fungal light sensor Vivid. Science.

[29] Kawano F., Suzuki H., Furuya A., Sato M. (2015). Engineered pairs of distinct photoswitches for optogenetic control of cellular proteins. Nat. Commun..

[30] Wang W., Li Y., Wang Y., Shi C., Li C., Li Q., Linhardt R.J. (2018). Bacteriophage T7 transcription system: An enabling tool in synthetic biology. Biotechnol. Adv..

[31] Shapiro T.H.S., Meyer A.J., Ellington A.D., Sontag E.D., Voigt C.A. (2014). A ‘resource allocator’ for transcription based on a highly fragmented T7 RNA polymerase. Mol. Syst. Biol..

[32] Kennedy M.J., Hughes R.M., Peteya L.A., Schwartz J.W., Ehlers M.D., Tucker C.L. (2010). Rapid blue-light–mediated induction of protein interactions in living cells. Nat. Methods.

[33] Schleif R. (2010). AraC protein, regulation of the l-arabinose operon in Escherichia coli, and the light switch mechanism of AraC action. FEMS Microbiol. Rev..

[34] Krajewski S.S., Narberhaus F. (2014). Temperature-driven differential gene expression by RNA thermosensors. Biochim. Biophys. Acta (BBA) Gene Regul. Mech..

[35] Liu M., Jin Z., Xiang Q., He H., Huang Y., Long M., Wu J., Huang C.Z., Mao C., Zuo H. (2024). Rational Design of Untranslated Regions to Enhance Gene Expression. J. Mol. Biol..

[36] Zhao P., Ma C., Xu L., Tian P. (2019). Exploiting tandem repetitive promoters for high-level production of 3-hydroxypropionic acid. Appl. Microbiol. Biotechnol..

[37] Chee W.K.D., Yeoh J.W., Dao V.L., Poh C.L. (2022). Highly Reversible Tunable Thermal-Repressible Split-T7 RNA Polymerases (Thermal-T7RNAPs) for Dynamic Gene Regulation. ACS Synth. Biol..

[38] Liu H., Zhang L., Wang W., Hu H., Ouyang X., Xu P., Tang H. (2023). An Intelligent Synthetic Bacterium for Chronological Toxicant Detection, Biodegradation, and Its Subsequent Suicide. Adv. Sci..

[39] Shen X., Zhou N., Liu S. (2012). Degradation and assimilation of aromatic compounds by *Corynebacterium glutamicum*: Another potential for applications for this bacterium?. Appl. Microbiol. Biotechnol..

[40] Cowles C.E., Nichols N.N., Harwood C.S. (2000). BenR, a XylS Homologue, Regulates Three Different Pathways of Aromatic Acid Degradation in Pseudomonas putida. J. Bacteriol..

[41] Liu D., Pakrasi H.B. (2018). Exploring native genetic elements as plug-in tools for synthetic biology in the cyanobacterium *Synechocystis* sp. PCC 6803. Microb. Cell Factories.

[42] Taggart J.C., Lalanne J., Li G. (2021). Quantitative Control for Stoichiometric Protein Synthesis. Annu. Rev. Microbiol..

[43] Li M., Wang J., Geng Y., Li Y., Wang Q., Liang Q., Qi Q. (2012). A strategy of gene overexpression based on tandem repetitive promoters in Escherichia coli. Microb. Cell Factories.

[44] Uchiyama T., Miyazaki K. (2010). Product-Induced Gene Expression, a Product-Responsive Reporter Assay Used To Screen Metagenomic Libraries for Enzyme-Encoding Genes. Appl. Environ. Microbiol..

[45] Brinkrolf K., Brune I., Tauch A. (2006). Transcriptional regulation of catabolic pathways for aromatic compounds in *Corynebacterium glutamicum*. Genet. Mol. Res. GMR.

[46] Baldanta S., Rodrigo G. (2025). Digitizing the Blue Light-Activated T7 RNA Polymerase System with a tet-Controlled Riboregulator. ACS Synth. Biol..

[47] Baumschlager A., Weber Y., Cánovas D., Dionisi S., Khammash M. (2026). Enhancing the performance of Magnets photosensors. Nat. Commun..

