# Supplementary material for: Design and Implementation of a Blue-Light-Controlled Gene-Switch System
Source: Molecules. 2026 Jun 10;31(12):2032. doi: 10.3390/molecules31122032 (PMC13304687; doi:10.3390/molecules31122032)
Supplement: Supplementary file 1 [file molecules-31-02032-s001.zip › molecules-4355961-supplementary.pdf]

Design and Implementation of a Blue-Light-Controlled Gene-Switch System

Chen Li, Yuan Shi, Xinyan Jiang, Bobo Zhao, Chen Zheng, Aowei Yang, Yao Wang, Junfeng Pan * and Xihui Shen *

Shaanxi Key Laboratory of Agricultural and Environmental Microbiology, College of Life Sciences, Northwest A&F University, Yangling 712100, China
* Correspondence: panjf@nwsuaf.edu.cn (J.P.); xihuishen@nwsuaf.edu.cn (X.S.)

Supplementary Table S1. Plasmids used in this study.

Plasmid	Description
pBAD-opto-T7RNAP(69)	Express N- and C-terminal fragments of T7NRAP by cleavage between residues 69 and 70. Amp^R
pBAD-opto-T7RNAP(302)	Express N- and C-terminal fragments of T7NRAP by cleavage between residues 302 and 303. Amp^R
pBAD-opto-T7RNAP(563)	Express N- and C-terminal fragments of T7NRAP by cleavage between residues 563 and 564. Amp^R
pKTT7-1-RBS1	Expression vector with engineered restriction sites for gene insertion. Kan^R
pKTT7-1-LacO-RBS1	The lactose operon element was incorporated into the construct based on pKTT7-1-RBS1. Kan^R
pKTT7-1-LacO-RBS2	Replace RBS1 with RBS2. Kan^R
pKTT7-1-LacO-RBS3	Replace RBS1 with RBS3. Kan^R
pKTT7-2-LacO-RBS1	Two T7 promoters are arranged in series. Kan^R
pKTT7-3-LacO-RBS1	Three T7 promoters are arranged in series. Kan^R
pKTT7-4-LacO-RBS1	Four T7 promoters are arranged in series. Kan^R
pKT-BenO-T7	Replace the lactose operon element with the element regulated by benzoic acid, BenO is located in front of the T7 promoter. Kan^R
pKT-T7-BenO	BenO is located behind the T7 promoter. Kan^R
pKT-2-BenO-T7	Two BenOs are arranged in series upstream of the T7 promoter. Kan^R

Amp^R: Ampicillin resistance; Kan^R: Kanamycin resistance.

Supplementary Table S2. Primers used in this study.

Primer	5'-3'	Purpose
Para-F	CCCGTTTTTTTGGGCTAGCAGGAGGAATTCACCGAGCTCTGGCATGCATCGATAGATGGTACCAAGAAACCAATTGTCCATATTGCAT	
Para-R	AAGCTTATCTATCGATGCATGCCACTCGAGGGTCATGAATTCCTCCTGCTAGCC	
p22aF	CTCGAGTGGCATGCATCGATAGATAAGCTTGGCTGTTTTGGCGGATGAGA	
p22aR	GTCGACATCTATCGATGCATGCCAGAGCTCGGTCATGAATTCCTCCTGCTAGCCC	
T7RNAP-N-F	GGCTAGCAGGAGGAATTCATGACCGAGCTCATGAACACGATTAACATCGCTAA	Used to construct the pBAD-opto-T7RNAP (302)
T7RNAP-N(302)-linker-R	ACCTCCAGAACCGCCCTTACTGTGAGTACGCACCAGC	
(302)nMag-linker-F	CGTACTCACAGTAAGGGCGGTTCTGGAGGTCACACTCTTTACGCCCCTG	
nMag-R	GGCCATCCGTCAGGATGGCCTTCTGTCGACTTAATGCCTTTCGGTTTCG	
pMag-F	GGCTAGCAGGAGGAATTCATGACCCTCGAGCACACTCTTTACGCCCCTG	
(303)pMag-linker-R	GCGCATCAGTGCTTTACCTCCAGAACCGCCTTCGGTTTCGCACTGGAAT	
T7RNAP-C(303)-linker-F	GGCGGTTCTGGAGGTAAAGCACTGATGCGCTACG	
T7RNAP-C -R	CTTCTCTCATCCGCCAAAACAGCCAAGCTTTTACGCGAACGCGAAGTC	
T7RNAP-N(69)-linker-R	GGCGTAAAGAGTGTGACCTCCAGAACCGCCGGCAGCGTTATCCGCAA	Used to construct the pBAD-opto-T7RNAP (69) or (563)
T7RNAP-C(70)-linker-F	CAGTGCGAAACCGAAGGCGGTTCTGGAGGTGCCAAGCCTCTCATCACTAC	
T7RNAP-N(563)-linker-R	GGCGTAAAGAGTGTGACCTCCAGAACCGCCAGGAAGCAAGTTAACCGC	
nMag-linker-F	GGCGGTTCTGGAGGTCACACTCTTTACGCCCCTG	
pMag-linker-R	ACCTCCAGAACCGCCTTCGGTTTCGCACTGGAAT	
T7+Lac-UP-F	TCTTCTGAGGTACCAGAAGGCCATCCTGACGGATGGCCTTTTTCACTGCCCGCTTTCCA	Used to construct the pKTT7-1-LacO-RBS1
T7+RBS1-UP-R	GTCGACGAGCTCGAATTCGGATCCCATGGTATATCTCCTTCTTAAAGTTAAACAAAATT	
T7+RBS1-DOWN-F	CTAGAAATAATTTTGTTTAACTTTAAGAAGGAGATATACCATGGGATCCGAATTCGAGC	
T7+Lac-	TGAAAAAGGCCATCCGTCAGGATGGCCTTCT	

DOWN-R	GGTACCTCAGAAGAACTCGTCAAGAAGG	
RBS2-UP-R	ACCTCCTCTATCGCGGAAATTGACAGGATCCTCTCGGAATTGTTATCCGCTCACAATTC	Used to construct the pKTT7-1-LacO-RBS2/RBS3
RBS2-DOWN-F	GATCCTGTCAATTTCCGCGATAGAGGAGGTAAAGCATATGGGATCCGAATTCGAGC	
RBS3-DOWN-F	ACACACACACACACACACACACACTAAGGAGGTCTATTCCATGGGATCCGAATTCGAGC	
RBS3-UP-R	TGTGTGTGTGTGTGTGTGTGTGGATCATCTAGAGGGGAATTGTTATCCGCTCACAATTC	
T7-2-DOWN-F	ATACTAGTTAATACGACTCACTATAGGCCTCTTAATACGACTCACTATAGGGGAATTGTGAGCGGATAACAATT	Used to construct the pKTT7-2/3/4-LacO-RBS1
T7-UP-R	AGGCCTATAGTGAGTCGTATTAACTAGTATTTCGCGGGATCGAGAT	
T7-3-DOWN-F	ATACTAGTTAATACGACTCACTATAGGCCTCTTAATACGACTCACTATAGGCCTCTTAATACGACTCACTATAGGGGAATTGTGAGCGGATAACAATT	
T7-4-DOWN-F	ATACTAGTTAATACGACTCACTATAGGCCTCTTAATACGACTCACTATAGGCCTCTTAATACGACTCACTATAGGCCTCTTAATACGACTCACTATAGGGGAATTGTGA	
2-BenO-UP-F	ACAATCCGGATAGGCAGAACTGGATAACACTCTGCACAATCCGGATATAATACGACTCACTATAGGCCT	Used to construct the pKT-2-BenO-T7
2-BenO-UP-R	CGGGCGTTTTTATTGGTGAGAATGGTACCTCAGAAGAACTCGTCAAGAAG	
2-BenO-DOWN-F	CTGAGGTACCATTCTCACCAATAAAAAACGC	
2-BenO-DOWN-R	TTCTGCCTATCCGGATTGTGCAGAGTGTTATCCAGTTCTGCCATTTCGCGGGATCGAGA	

Supplementary Table S3. Genetic parts used in this study.

Name	5'-3'
P_{araBAD}	AAGAAACCAATTGTCCATATTGCATCAGACATTGCCGTCACTG
	CGTCTTTTACTGGCTCTTCTCGCTAACCAAACCGGTAACCCCG
	CTTATTAAAAGCATTCTGTAACAAAGCGGGACCAAAGCCATG
	ACAAAAACGCGTAACAAAAGTGTCTATAATCACGGCAGAAAA
	GTCCACATTGATTATTTGCACGGCGTCACACTTTGCTATGCCAT
	AGCATTTTTATCCATAAGATTAGCGGATCCTACCTGACGCTTTT
	TATCGCAACTCTCTACTGTTTCTCCAT
Ter	AGAAGGCCATCCTGACGGATGGCCTTTT
nMag	CACACTCTTTACGCCCCTGGAGGATACGACATTATGGGATATTT
	GGATCAGATTGGGAACCGCCCAAACCCTCAGGTCGAACTGGG
	GCCTGTGGACACGTCATGTGCCCTGATCCTGTGCGATCTGAAG
	CAAAAGGACACTCCGATCGTCTACGCCTCGGAAGCCTTCTTGT
	ATATGACCGGATACAGCAATGCAGAGGTGCTCGGCAGGAACT
	GCAGATTCCTGCAGTCCCCCGACGGGATGGTGAAACCAAAGT
	CGACTCGCAAATATGTGGACTCGAACACGATCAACACCATCCG
	GAAGGCCATCGACCGGAACGCCGAGGTCCAGGTGGAGGTGG
	TCAACTTTAAGAAGAACGGCCAGCGGTTCGTGAACTTTCTGA
	CCATCATTCCGGTCCGGGATGAAACCGGAGAGTACAGATACTC
	CATGGGATTCCAGTGCGAAACCGAA
pMag	CACACTCTTTACGCCCCTGGAGGATACGACATTATGGGATATTT
	GCGGCAGATTAGGAACCGCCCAAACCCTCAGGTCGAACTGGG
	GCCTGTGGACACGTCATGTGCCCTGGTCCTGTGCGATCTGAAG
	CAAAAGGACACTCCGGTGGTCTACGCCTCGGAAGCCTTCTTG
	TATATGACCGGATACAGCAATGCAGAGGTGCTCGGCAGGAAC
	TGCAGATTCCTGCAGTCCCCCGACGGGATGGTGAAACCAAAG
	TCGACTCGCAAATATGTGGACTCGAACACGATCAACACCATGC
	GGAAGGCCATCGACCGGAACGCCGAGGTCCAGGTGGAGGTG
	GTCAACTTTAAGAAGAACGGCCAGCGGTTCGTGAACTTTCTG
	ACCATGATTCCGGTCCGGGATGAAACCGGAGAGTACAGATAC
	TCCATGGGATTCCAGTGCGAAACCGAA
P_{T7}	TAATACGACTCACTATAGG
BenO	GGCAGAACTGGATAACACTCTGCACAATCCGGATA
BenR	ATGGAAAGCCGCCTGCTGAGCGAGCGAAGTAGCGTGTTTCAC
	CACGCCGACCCTTATGCTGTGTCCGATTATGTGAACCAGCATG
	TAGGCCAGCATTGCATCGGTCTGTCCCGTACCACCCATCCCCA
	GGCTAGCCTCAGCCACCGCAAGTTTGCCGAGCTCGACCTGTG
	CCGTATCAGCTATGGCGGCAGTGTTCGCGTGACTTCTCCAGCG
	CTGGAAACCATTTATCACCTGCAAGTGTTGCTCAACGGCAACT
	GCCTGTGGCGTGGGCACAAGCACGAGCAGCACCTGGTGCCG
	GGCGAGCTGCTGCTGATCAACCCGGACGACCCGGTTGACCTG
	ACCTATTCGGAAGACTGCGAGAAGTTCATCCTCAAGGTGCCG
	ACCCGGCTGCTGGACTCGATCTGCGATGAACAGCGCTGGCAG

	CGGCCTGATGGGGGCGTGCGGTTCTTGCGCAATCATTACCGGCTGGATGAGCTGGATGGCTTCGTCAACCTGCTGGCCATGGTTTGTCATGAGGCGGAAGTGAGCGAGTCGCTGCCCAGGGTGCAGGGGCACTACAGCCAAATTGTCGCCAGCAAGTTGCTGACCCTGATGACCACCAATATCCGCCGGGAGAGCCTGAGTGCACCGCAGGCCGGCCTTGAGCGCATTCTCGATTACATAGAGCGCAACCTGAAGTTGGAGCTGTCGGCCGAGGTGCTGGCAGAGCAGGCCTGCATGAGTTTGCGTTCGCTGTATGCGCTGTTTGAACAGCACCTGAGCACCACGCCCAAGCATTACGTGCGCCAGCGCAAGCTTGAGCGGGTGCATGCGTGCCTGAGCGACCCGACTTGCGGTGTGCGCAGTGTGACCGAACTTGCCCTGGATTACGGCTTCTTTCATTTAGGCCGGTTTTCCGAGGTGTATCGGCAGCAATTTGGCGAGTTGCCTTCGCAGACGTTCAAGCGTCGGGGGTGA
RBS1	CCTCTAGAAATAATTTTGTTTAACTTTAAGAAGGAGATATACC (TIR = 1)
RBS2	GAGAGGATCCTGTCAATTTCCGCGATAGAGGAGGTAAAGCAT (TIR = 0.78)
RBS3	CCTCTAGATGATCCACACACACACACACACACACACACACACTAAGGAGGTCTATTCC (TIR = 2.15)

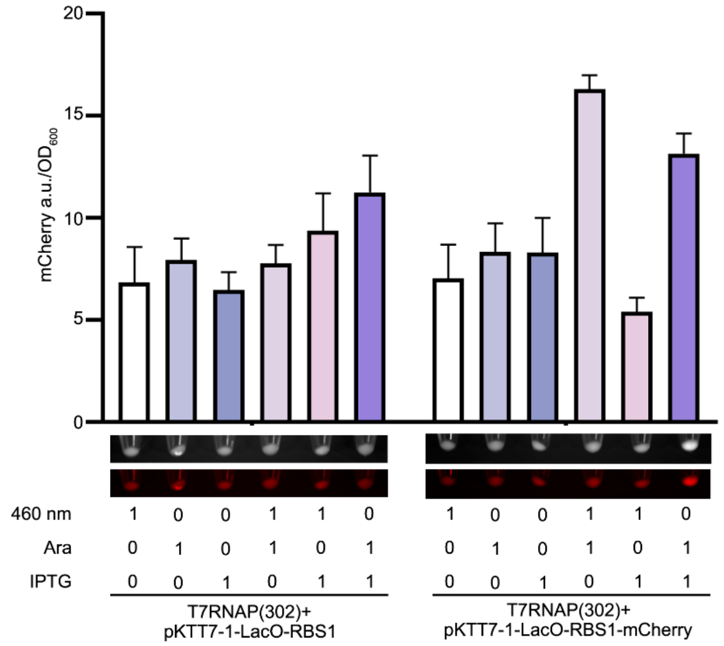

Supplementary Figure S1. Full set of experimental controls for the tripartite inducible system. Fluorescence output (mCherry a.u. / OD_{600}) under all 8 possible combinations of inducer inputs (460 nm blue light, L-arabinose, IPTG), as indicated by the binary matrix below each bar group. Left panel: Reporter plasmid without mCherry (pKTT7-1-LacO-RBS1); Right panel: Reporter plasmid with mCherry (pKTT7-1-LacO-RBS1-mCherry). Each bar represents mean ± SEM from n = 3 biological replicates.

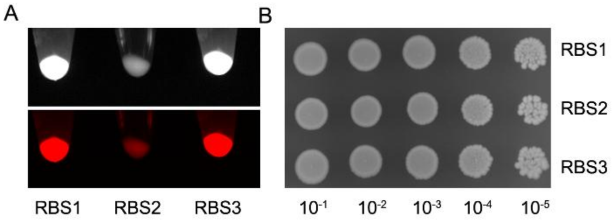

Supplementary Figure S2. Evaluation of mCherry expression driven by different RBS sequences. A Fluorescence imaging of mCherry expression in *E. coli* K12 strains harboring RBS1, RBS2, or RBS3. Increased red fluorescence intensity indicates enhanced translation efficiency. B Spot assay under uninduced conditions to assess the baseline impact of different RBSs on cell growth or viability. Serial dilutions (10^{-1} to 10^{-5}) were plated to visualize colony formation and density.

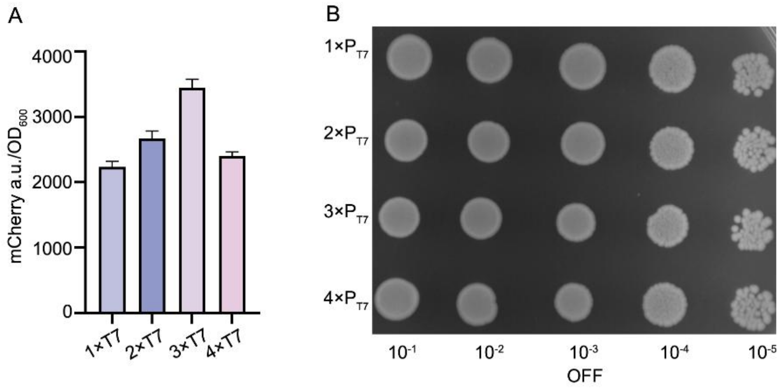

Supplementary Figure S3. Correlation between mCherry expression and ccdB-mediated growth inhibition under varying T7 promoter copy numbers.

(A) Fluorescence output (mCherry a.u. / OD_{600}) under full induction (Ara + blue light + IPTG) for constructs with 1×, 2×, 3×, or 4× T7 promoters. Each bar represents mean ± SEM. from n = 3 biological replicates.

(B) Bacterial growth on solid LB plates after 24 h incubation, serially diluted from 10^{-1} to 10^{-5}. Growth inhibition correlates with increased T7 promoter copy number, consistent with higher mCherry expression in panel A. This confirms that ccdB-mediated growth suppression serves as a reliable proxy for gene expression level. OFF: uninduced.

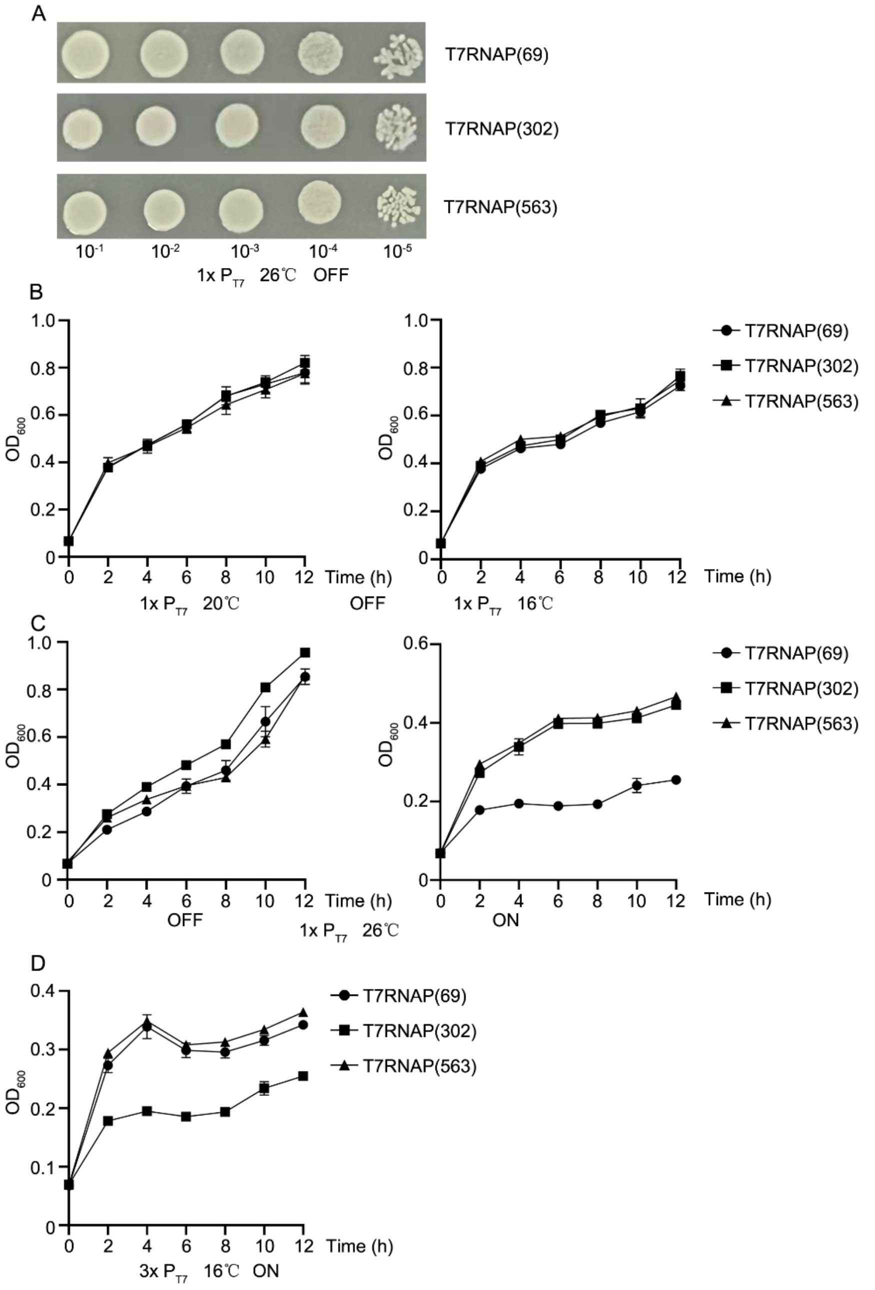

Supplementary Figure S4. Effect of T7RNAP cleavage site position on system expression. A. Spot assay comparing bactericidal efficacy of T7RNAP variants with cleavage sites at positions 69, 302, and 563. Serial dilutions (10^{-1} to 10^{-5}) are shown for each construct. B. Growth curves of bacterial cultures expressing T7RNAP variants with cleavage sites at positions 69, 302, and 563 at 20°C (left) and 16°C (right). No inducers were added during the experiment. C. Growth curves of bacterial cultures expressing T7RNAP variants with cleavage sites at positions 69, 302, and 563 at 26°C. Uninduced (left), induced (right). D. Growth curves of bacterial cultures expressing T7RNAP variants with cleavage sites at positions 69, 302, and 563 driven by three tandem T7 promoters at 16°C. OFF: uninduced. ON: induced.

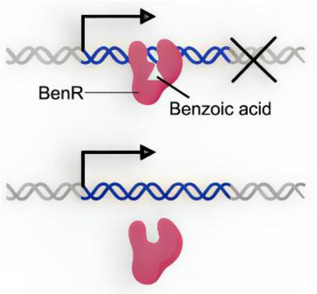

Supplementary Figure S5. **Schematic representation of the benzoate-negative regulatory module.** Top: In the presence of benzoic acid, the regulator protein (BenR) binds to the operator sequence on the DNA, physically blocking the progression of RNA polymerase (indicated by the black arrow) and repressing downstream gene expression. Bottom: In the absence of benzoic acid, the BenR protein dissociates from the DNA, allowing RNA polymerase to bind to the promoter and proceed with active transcription.

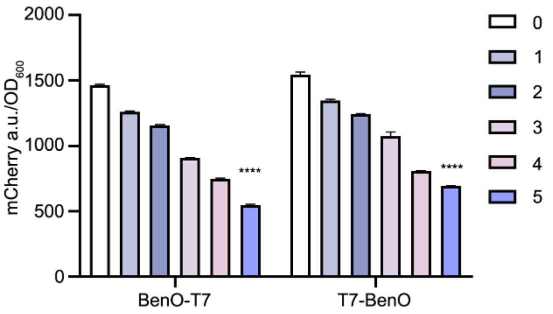

Supplementary Figure S6. Dose-dependent response of two engineered benzoate-responsive systems, BenO-T7 (BenO operator upstream of T7 promoter) and T7-BenO (T7 promoter upstream of BenO operator), to increasing concentrations of benzoic acid (0–5 mM). mCherry fluorescence (a.u./OD_{600}) was measured as a reporter of promoter activity. Data represent mean ± SEM (n = 3). ****: $p < 0.0001$ compared to 0 mM benzoate within the same system (two-way ANOVA).
